# Krill oil supplementation alleviates clinical symptoms and improves intestinal barrier function with transcriptomic associations to cell adhesion molecule pathways in a mouse model of spontaneous chronic colitis

**DOI:** 10.3389/fnut.2026.1848942

**Published:** 2026-08-04

**Authors:** Yingying Liu, Ainsley M Robinson, Kulmira Nurgali, Xiao Qun Su

**Affiliations:** 1 Institute for Health & Sport, Victoria University, Melbourne, VIC, Australia; 2 Department of Medicine Western Health, The University of Melbourne, Melbourne, VIC, Australia; 3 Regenerative Medicine and Stem Cells Program, Australian Institute for Musculoskeletal Science (AIMSS), Melbourne, VIC, Australia

**Keywords:** cell adhesion molecule (CAM) pathways, colonic permeability, dexamethasone, IBD, krill oil, *Winnie* mice

## Abstract

**Background:**

Conventional therapies for inflammatory bowel disease (IBD) are associated with significant adverse effects, highlighting the need for safer and effective alternatives. Krill oil (KO), a rich source of long-chain omega-3 polyunsaturated fatty acids (PUFAs) and astaxanthin, has shown therapeutic potential in several disorders but remains understudied in chronic colitis. This study evaluates the potential therapeutic efficacy of KO, alone or in combination with dexamethasone (DEX), in a spontaneous chronic colitis mouse model that closely mimics human IBD. The molecular mechanisms underlying the health benefits of KO were also explored.

**Methods:**

Four groups of homozygous *Winnie* mice (*Win*/*Win*) received 10% KO, 20 ng/g DEX, KO + DEX, or saline (sham) for 28 days. Heterozygous littermates (*Win*/*Wt*) were used as controls (*n =* 10 per group). Disease activity, colonic histopathological changes, epithelial barrier integrity, and underlying molecular mechanisms were evaluated.

**Results:**

Treatments with KO, DEX, and KO + DEX significantly reduced disease severity and mucosal injury. Restoration of colonic permeability was observed across all treatment groups, with KO + DEX and DEX treatments notably enhancing the expression of tight junction proteins. Transcriptomic profiling identified changes in epithelial barrier-related and cell adhesion molecules (CAM)-related genes following KO treatment, including higher expression of *Cldn1*, *Cldn4*, *Galnt3* and lower expression of *Icam1*, *Selp*, *Sele*, *Ptprc*. Combination therapy with KO and DEX was associated with gene expression patterns related to epithelial polarity and extracellular matrix anchoring (*Ctnnb1*, *Dsg2*, *Muc4*, *Itga8*), as well as lower expression of CAM-related genes implicated in immune cell recruitment.

**Conclusion:**

KO treatment showed promising therapeutic benefits, with some being comparable to DEX, and enhanced efficacy when combined with DEX, in a mouse model of spontaneous chronic colitis by restoring epithelial barrier integrity and suppressing CAM-mediated immune cell recruitment.

## 1 Introduction

Inflammatory bowel disease (IBD), encompassing Crohn’s disease (CD) and ulcerative colitis (UC), is a chronic and relapsing inflammatory condition of the gastrointestinal (GI) tract that affects approximately 4.9 million individuals worldwide (1, 2). CD is characterized by transmural inflammation and discontinuous skip lesions throughout the GI tract, whereas UC presents as continuous mucosal and submucosal inflammation confined to the colon and rectum (3). Despite advances in pharmacological therapies, including corticosteroids, aminosalicylates, antibiotics, immunosuppressants, and biologics, there is no curative treatment available, and many interventions have limited application due to their adverse effects or short-term efficacy (4, 5). Corticosteroids, such as dexamethasone (DEX), remain as a first-line anti-inflammatory option (6, 7), but their use is associated with notable adverse effects. Short-term administration may cause insomnia, weight gain, and mood disturbances, while prolonged exposure increases the risk of bone loss, infections, and mortality (8). These limitations have prompted growing interest in nutraceuticals as safer, adjunctive therapies for IBD.

Diet has been established as a modifying factor in the prevention and treatment of CD and UC (9). Studies have shown a significant inverse relationship between dietary long-chain omega-3 polyunsaturated fatty acids (LC n-3 PUFAs), eicosapentaenoic acid (EPA, 20:5n-3) and docosahexaenoic acid (DHA, 22:6n-3) and the risk of developing UC (10). LC n-3 PUFAs derived from fish oil have demonstrated beneficial effects on IBD, including a reduction in the need for corticosteroid therapy (11). Krill oil (KO) contains high levels of LC n-3 PUFAs bound to phospholipids instead of triglycerides as in the fish oil (12). Studies have suggested that the bioavailability of phospholipid-bound n-3 PUFA is higher than those bound to triglycerides, hence may lead to more health benefits (13). In addition, KO also contains astaxanthin, vitamins A and E, and flavonoids (2).

The unique biochemical properties of KO implicate it may be a promising candidate for IBD therapy. Few studies have found that KO can attenuate acute colitis and reduce intestinal inflammation in animal models induced by chemicals, parasites, or bacteria (2). However, its efficacy in chronic colitis remains poorly understood. Given the limitations of chemically-induced experimental models, which are affected by various environmental, genetic, and immunological factors and do not fully replicate the complex pathogenesis of human IBD (14), we investigated the efficacy of KO supplementation in comparison with a clinically used therapeutic agent, DEX, in a mouse model of spontaneous chronic colitis, closely resembling human IBD.

*Winnie* mice (C57BL/6 background) are a well-established model of chronic colitis (15). In these mice, spontaneous colitis arises due to a primary defect in the intestinal epithelium caused by a mutation in the *Muc2* mucin gene, which effectively mirrors pathophysiological mechanisms and clinical manifestations of human IBD (16). MUC2 is the major secreted mucin forming the intestinal mucus barrier, and its proper folding and O-glycosylation are essential for maintaining epithelial protection (17–21). *Winnie* mice develop intestinal inflammation starting around 5–6 weeks of age, progressing to severe colitis by 16 weeks, accompanied by abnormal MUC2 biosynthesis, altered mucus layer structure, increased intestinal permeability, and heightened susceptibility to luminal inflammation-inducing toxins (20).

This study aimed to (i) evaluate the therapeutic efficacy of dietary KO supplementation in the *Winnie* mouse model of chronic colitis by assessing disease activity, colonic mucosal architecture, intestinal permeability, epithelial barrier integrity, and unravel associated molecular mechanisms; and (ii) compare KO supplementation with DEX monotherapy and determine the potential complementary effects of combined KO + DEX treatment. This study provides pre-clinical evidence for the efficacy and mechanisms of KO supplementation in a spontaneous chronic UC model and determine its potential as an alternative therapeutic agent or a complement to corticosteroid treatment for IBD.

## 2 Materials and methods

### 2.1 Animals

Twelve-week-old male and female *Winnie* mice were obtained from the Victoria University Werribee Animal Facility (Melbourne, Australia). Homozygous *Winnie* mice (*Win*/*Win*) were randomly assigned to experimental groups (KO, DEX, KO + DEX, sham; *n =* 10/group), while heterozygous littermates (*Win*/*Wt*) served as controls (*n =* 10, see Supplementary Figure S1). Each experimental group initially had 10 mice (5 males and 5 females). The final sample sizes varied across analyses as indicated in the respective figure legends. All mice were housed under controlled conditions (22 °C, 12-h light/dark cycle) with unrestricted access to food and water. Following a 3-day acclimatization period, mice received treatments for 28 days and were euthanized on day 29 via intraperitoneal injection of 10% pentobarbital (100 mg/kg) followed by cervical dislocation. All procedures were adhered to the Australian Code of Practice for the Care and Use of Animals for Scientific Purposes and were approved by the Victoria University Animal Ethics Committee (AEC 22/018).

### 2.2 Treatments

KO (Swisse Wellness Pty Ltd., Victoria, Australia), containing 21% EPA and 10% DHA, was incorporated into the standard rodent diet (at a concentration of 10%). The 10% dietary KO concentration and treatment protocol were selected based on our previous studies that evaluated the therapeutic efficacy of various KO concentrations without observed adverse effects in colorectal cancer model (22). Control, DEX, and sham-treated groups received the standard rodent diet. DEX (20 ng/g body weight; Sigma Aldrich, Victoria, Australia) was dissolved in saline and administered via intraperitoneal injection once daily. The dose and administration route of DEX used in this study was based on the study by Das et al. (23) which demonstrated alleviation of endoplasmic reticulum stress and intestinal inflammation in *Winnie* mice without causing significant toxicity. Control, sham, and KO groups received equivalent volumes of saline (max 200 μL) as vehicle control. Mice were treated for 28 days and monitored daily for rectal prolapse, diarrhea, and body weight. Animals exhibiting ≥15% weight loss or rectal prolapse were humanely euthanized.

### 2.3 Assessment of disease activity

The body weight, feces viscosity, stool consistency, occult/gross bleeding, and prolapse conditions of mice from each group were monitored daily throughout the 28-day treatment period. The disease activity index (DAI) score was recorded according to the scoring criteria listed in Table 1. After culling, colons were excised and their length (from cecum to anus) was measured immediately by placing the colon alongside a ruler. Subsequently, fresh distal colon sections were collected for histological and immunohistochemical (IHC) analyses. Fecal water content was determined weekly by comparing wet and dry weights of freshly collected feces.

**Table 1:** Evaluation criteria of disease activity index (DAI).

DAI score	Weight loss (%)	Stool consistency	Occult/gross bleeding
0	None	Normal	Negative
1	1–5	Loose stool	Negative
2	5–10	Loose stool	Hemoccult positive
3	10–15	Diarrhea	Hemoccult positive
4	10–15	Diarrhea	Gross bleeding

### 2.4 Histology

Distal colon tissues were pinned flat with the mucosal side facing up and fixed overnight at 4 °C in a 10% (w/v) neutral-buffered formalin solution. Subsequently, the fixed tissues were dehydrated through a series of graded ethanol solutions (70, 80, 90, 95, and 100%, each for 1 h), cleared in xylene, and embedded in paraffin. Tissues were sectioned at a thickness of 8 μm using a Microtome Hyrax M40 (Carl Zeiss, Germany). Colon sections were deparaffinized and rehydrated through graded ethanol concentrations. For hematoxylin and eosin (H&E) staining, sections were immersed in xylene (3 × 5 min), followed by immersion in 100% ethanol (1 min), 90% ethanol (1 min), and 70% ethanol (1 min). The sections were then rinsed in tap water (30 s), stained with hematoxylin (Sigma–Aldrich) for 1 min, rinsed in tap water, immersed in Scott’s tap water substitute (1 min), and stained with eosin (Sigma–Aldrich) for 3 min. After rinsing in tap water, the sections were immersed in 100% ethanol (2 × 1 min) and xylene (5 min) and finally mounted on glass slides using distyrene plasticizer xylene (DPX) mounting medium (Sigma–Aldrich). For Alcian blue staining, sections underwent a similar process: immersion in xylene (3 × 5 min), followed by immersion in 100% ethanol (1 min), 90% ethanol (1 min), and 70% ethanol (1 min). After rinsing in tap water (30 s), the sections were stained with Alcian blue (Sigma–Aldrich) for 15 min, rinsed in tap water, and stained with eosin (Sigma–Aldrich) for 2 min. The sections were then rinsed in tap water, immersed in 100% ethanol (2 × 1 min each) and xylene (5 min), and mounted on glass slides using DPX mounting medium for subsequent imaging.

A histological grading system was employed to evaluate gross morphological damage using the following parameters: aberrant crypt architecture (score range 0–3), changes in crypt length (0–3), crypt abscesses (0–3), leukocyte infiltration (0–3), epithelial damage (0–3), and ulceration (0–3) (24).

### 2.5 Colonic permeability

Intestinal permeability was assessed using FITC-Dextran 4 kDa (FD4) (60,842,468, Merck, Australia) prior to treatments (day 0) and at day 29. Prior to FD4 administration, mice were fasted for 4 h. FD4 (100 mg/mL in distilled water) was administered via oral gavage using a plastic feeding tube. After 4 h, approximately 200 μL of blood sample was collected by submandibular vein puncture and centrifuged at 3000 rpm for 15 min at 4 °C. Then, 100 μL of the supernatant was placed in a 96-well plate and FITC (485 nm/535 nm) fluorescence was measured using the Varioskan Flash Multimode Reader (Thermo Fisher Scientific, Waltham, Massachusetts, USA). FD4 concentrations were determined from standard curves created by serial dilutions of FD4.

### 2.6 Immunohistochemistry

Distal colon tissues were harvested in Krebs solution, cut along the mesenteric border, and flushed clear of fecal content. The tissues were pinned flat with the mucosal side facing up in Sylgard coated Petri dishes and fixed overnight with Zamboni’s fixative (2% formaldehyde containing 0.2% picric acid) at 4 °C. The fixative was removed through 3 × 10 min washes with dimethyl sulfoxide (DMSO; Sigma–Aldrich, Sydney, Australia), followed by 3 × 10 min washes with PBS. Subsequently, the tissues were placed in 30% sucrose-PBS for 24 h, embedded in optimal cutting temperature (OCT) compound (Tissue Tek, CA, USA), frozen in liquid nitrogen-cooled isopentane, and stored at −80 °C until cryo-sectioning (20 μm) onto glass slides using a cryostat (Leica CM 1950, Biosystems, Germany).

For IHC, samples were incubated for 1 h at room temperature in 10% normal donkey serum (NDS; Merck Millipore, Australia) in phosphate-buffered saline-0.1% Tween 20 (PBST). Preparations were then washed 3 × 10 min with PBST and incubated with primary antibodies in PBST + 2% NDS overnight at 4 °C in an enclosed moist environment. The next day, cross-sections were washed with PBST (3 × 10 min) and incubated with fluorophore-conjugated secondary antibodies in PBST + 2% NDS for 2 h at room temperature. Tissues were then washed in PBST (4 × 10 min). All sections were stained with 300 nM 4′, 6′-diamidino-2-phenylindole dihydrochloride (DAPI, Invitrogen, USA) to label cell nuclei and cover slipped with fluorescent mounting medium (S3023, DAKO, Australia) for imaging.

### 2.7 RNA extraction and high-throughput RNA sequencing

Total RNA was extracted from distal colons of *Winnie* mice (*n =* 6) using the RNeasy Lipid Tissue Mini Kit (Qiagen, Germany), following manufacturer’s protocols. RNA integrity was assessed using the Tapestation 4,150 system (Agilent, USA), while purity (260/280 and 260/230 ratios >1.8) and concentration were determined using a Nanodrop spectrophotometer (Denovix Inc., USA). mRNA sequencing was conducted by Micromon Genomics (Monash University, Melbourne, Australia) using the MGISEQ2000RS platform. Pre-sequencing quality control included Qubit RNA BR assay and polyA purification. Libraries were prepared using MGIEasy V2 chemistry and sequenced with ≥400 M raw paired-end 100 bp reads per lane. Data processing employed nf-core/rnaseq v3.10.1 with the GRCm39.release-109 genome. Read counts were quantified via FeatureCounts and normalized using Degust web platform. Differential expression analysis was performed using EdgeR (v3.42.2), with significance defined as a false discovery rate (FDR) < 0.05 and log2 fold change (log2FC) ≥ ±0.585.

### 2.8 Differential expression and visualization of genes associated with epithelial barrier function

To investigate molecular alterations associated with epithelial barrier integrity, we used the Molecular Signatures Database (MSigDB v2025.1) for gene sets relevant to barrier function. Keywords searched include “tight junction,” “epithelial barrier,” “intestinal permeability,” and “adherence junction,” spanning curated collections (C2) and hallmark gene sets (H). Retrieved gene sets were manually curated to remove redundancy and compiled into a unified barrier-associated gene set (Supplementary File 1: Barrier-associated gene set). We additionally assembled a list of 100 core structural proteins implicated in epithelial and gut permeability (Supplementary File 2: Barrier-associated structural genes). These proteins were selected based on their established roles in junctional architecture (e.g., tight junctions, adherens junctions, desmosomes), cytoskeletal anchoring, and transmembrane scaffolding relevant to barrier regulation (25).

Differential expression analysis was performed using EdgeR (v3.42.2), with FDR < 0.05 and |log₂FC| ≥ 0.585. Differentially Expressed Genes (DEGs) were defined as the intersection between DEGs and the curated barrier gene set. Expression profiles of structural protein-coding genes associated with epithelial and gut barrier function were visualized across colonic samples using the *pheatmap* (v1.0.13) in R (v4.3.1). Raw counts were normalized to count per million (CPM), scaled to *Z*-scores, and plotted as heatmaps. Hierarchical clustering of both genes and samples was performed using Euclidean distance to assess expression patterns and sample stratification.

Functional enrichment analysis of barrier-related DEGs was performed using the enrichKEGG function from the clusterProfiler (v4.17.0), based on overrepresentation analysis against the Kyoto Encyclopedia of Genes and Genomes (KEGG) database. Pathways with adjusted *p* value < 0.05 were considered significantly enriched. Enrichment results were visualized using the dotplot function.

For pathway-level visualization, the most significantly enriched KEGG pathway was rendered using the Pathview package (v1.49.0), incorporating gene-level log₂ fold changes to highlight upregulated and downregulated components within the pathway architecture. Expression levels and log₂ fold changes were visualized across treatment groups using ggplot2 package (v3.4.4).

### 2.9 Imaging and quantitative analysis

H&E- and Alcian blue-stained colon sections were imaged at 20x magnification using a BX53 Olympus Fluorescence Microscope (Olympus Imaging, Sydney, Australia) to assess gross morphological damage. On average, five areas of 1 mm^2^ per section were evaluated using histological grading system (total area 5 mm^2^). Immunolabeled specimens were visualized and imaged using a Leica STELLARIS 5 inverted confocal laser-scanning microscope (Leica Microsystems, Wetzlar, Germany) with DAPI, Alexa 488, and Alexa 594 filters. Images (1,024 × 1,024 pixels) were captured using 20x (dry) lenses and 63x (oil) lenses, with Z-series images obtained at a total thickness of 22 μm. Immunoreactivity (IR) for CD45 (Ab10558, 1:500, Abcam, MA, USA), Claudin-1 (Ab15098, 1:500, Abcam, MA, USA), and EpCAM (TF 14579185, 1:500, Thermo Fisher Scientific, MA, USA) was analyzed in cross-sections of the colon and quantified as a percentage of the specific fluorescence area relative to the total 2 mm^2^ area of the colonic mucosa. All images were acquired with equal exposure and acquisition times, calibrated to a standard minimum baseline fluorescence, and converted to binary for density analysis. Data were normalized and presented as percentages due to variability in colon sizes among groups. All analyses were conducted blindly using ImageJ software (National Institute of Health, Bethesda, MD, USA).

### 2.10 Statistical analysis

Data were assessed using one-way ANOVA with Tukey *post hoc* tests for multiple group comparison. Analyses were performed using GraphPad Prism 10 software (Graph Pad Software Inc., San Diego, CA, USA). Sex-stratified analyses of DAI scores were performed using one-way ANOVA and Mann–Whitney *U* tests with Bonferroni correction. Data are presented as mean ± standard error of the mean (SEM). Value differences were considered statistically significant when *p <* 0.05.

## 3 Results

### 3.1 Krill oil treatment improved clinical symptoms in *Winnie* mice

**Figure 1:**
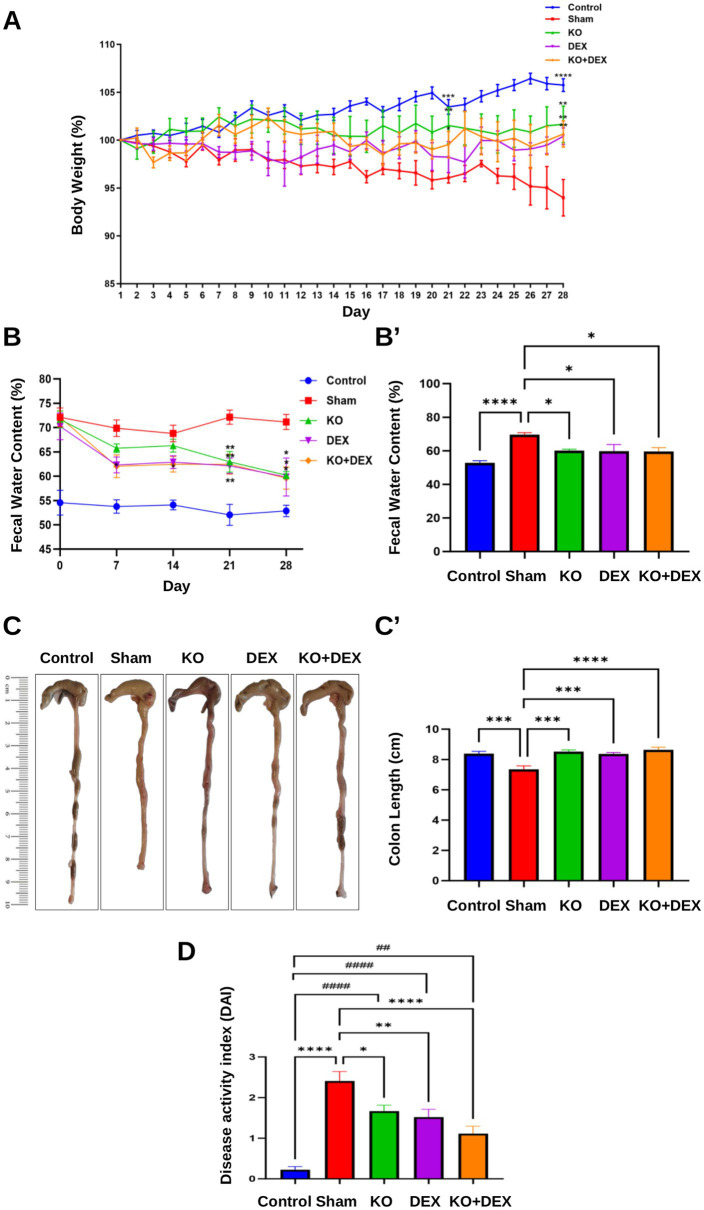
The effects of KO, DEX, and KO + DEX treatments on the clinical symptoms of colitis in *Winnie* mice. **(A)** Changes in body weight (%) were monitored over a period of 28 days (*n* = 9/group). **(B,B’)** Fecal water content (the difference between wet and dry weight) was calculated as a percentage of the wet weight of fresh fecal pellets and fecal water retention was measured over the 28-day treatment period at days 0, 7, 14, 21, and 28 (*n* = 8/group). **(C,C’)** Visual representation and quantification of colon length on day 29 (*n* = 7/group). **(D)** DAI scores were assessed on day 29 (*n* = 8/group). Data expressed as mean ± SEM, *p < 0.05, **p < 0.01, ***p < 0.001, ****p < 0.0001 compared with *Win/Win* sham-treated mice; ^#^*p* < 0.05 compared with *Win/Wt* control mice.

To evaluate the systemic effect of KO, body weights were recorded daily over the 28 days of treatment period, and the changes were calculated as a percentage relative to the initial body weight (Figure 1A). Although body weights fluctuated during the treatment period, *Win*/*Win* sham-treated mice showed a progressive decrease in their weight (93.98 ± 1.91%, *n =* 9) compared to control mice (105.75 ± 0.67%, *n =* 9, *p* < 0.0001) by day 28. The body weight of KO-treated mice was similar to those treated with DEX, and combined KO + DEX (KO 101.65 ± 1.92%; DEX: 100.40 ± 0.88%; KO + DEX: 100.65 ± 1.38% at day 28, *n =* 9/group), and they were all significantly higher than the body weight of *Win*/*Win* sham-treated mice (*p* < 0.01 for all). No statistically significant differences were observed between the three treatment groups and the *Win/Wt* control group.

Diarrhea was confirmed by measuring the fecal water content in freshly collected feces from all groups. Over the 28-day treatment period, fecal water content was consistently higher in *Win/Win* sham-treated group when compared to control mice (Figure 1B). Sham-treated mice exhibited diarrhea, contained soft fecal masses and had higher fecal water content (69.52 ± 1.35%) compared to control mice, which produced firm fecal pellets on day 28 (fecal water content: 52.87 ± 1.18%, *p* < 0.0001, *n =* 8/group, Figure 1B’). Treatments with KO, DEX, and KO + DEX improved fecal viscosity and resulted in lower fecal water content in *Win*/*Win* mice (KO: 60.2 ± 0.75%; DEX: 59.83 ± 3.92%; KO + DEX: 59.61 ± 2.28%, *p* < 0.05 for all, *n =* 8/group). Although fecal water content in KO-, DEX-, and KO + DEX-treated mice remained higher than in controls, all three treatments showed a clear downward trend over time when compared with sham-treated *Win*/*Win* mice.

The length of the excised colons was measured after 28 days of treatment (Figures 1C,C’). Colons from sham-treated mice were shorter (7.34 ± 0.24 cm, *n =* 7) compared to those from control mice (8.39 ± 0.15 cm, *p* < 0.001, *n =* 7, Figure 1C’). Treatments with KO (8.51 ± 0.12 cm, *p* < 0.001), DEX (8.37 ± 0.11 cm, *p* < 0.001), and KO + DEX (8.64 ± 0.19 cm, *p* < 0.0001) (*n =* 7/group) improved colon length when compared to sham-treated mice, achieving the length similar to those of control mice. No significant differences were observed between KO, DEX, and KO + DEX treatment groups.

*Win*/*Wt* control mice did not display any symptoms of inflammation or disease activity (Figure 1D). In contrast, sham-treated *Win*/*Win* mice displayed colitis symptoms, including chronic diarrhea and body weight loss (DAI score: control: 0.22 ± 0.08; sham: 2.41 ± 0.23, *p* < 0.0001, *n* = 8/group). Notably, two mice from the *Win*/*Win* sham group developed severe colitis, which resulted in rectal prolapse and rectal bleeding by the age of 16 weeks. However, in other groups, no blood in the stool was observed. Treatments with KO, DEX, and the combination of KO + DEX markedly alleviated clinical symptoms of diarrhea, rectal prolapse, and bleeding in *Win/Win* mice (DAI score—KO: 1.67 ± 0.15, *p* < 0.05; DEX: 1.52 ± 0.19, *p* < 0.01; KO + DEX: 1.11 ± 0.18, *p* < 0.0001, *n =* 8/group). However, the DAI score of KO-treated mice remained higher than those of the control group (*p* < 0.0001). Sex-stratified analyses of DAI scores revealed significant group differences in both male and female mice (*p* < 0.001 for both). No significant sex differences were observed within any treatment group following Bonferroni correction, indicating that treatment effects were consistent across sexes (*p* > 0.05 for all, data not shown).

### 3.2 Krill oil treatment mitigated colonic mucosal damage in *Winnie* mice

**Figure 2:**
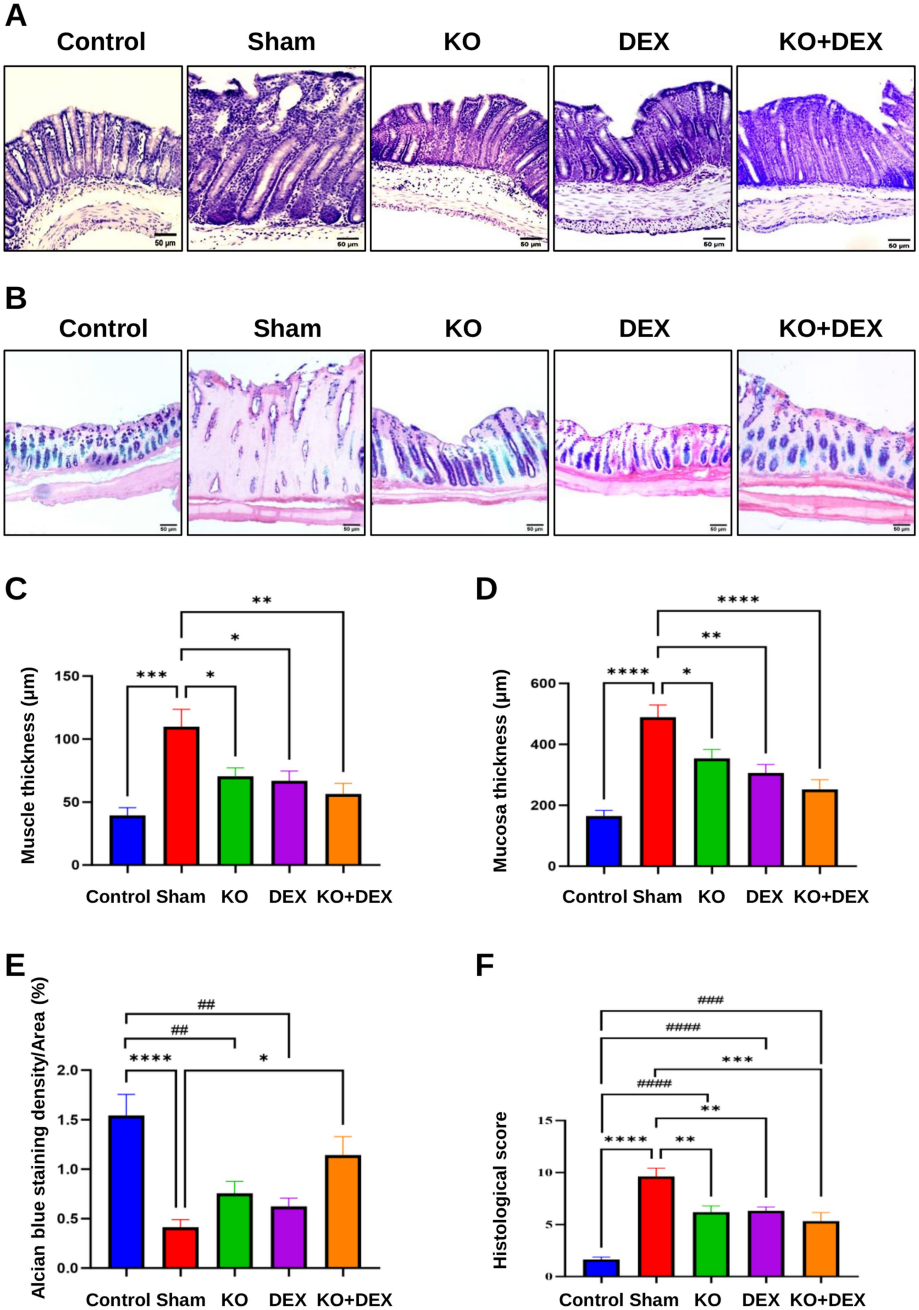
The effects of KO, DEX, and KO + DEX treatments on gross morphological damage in *Winnie* mice. Representative images of H&E-stained **(A)** and Alcian blue-stained **(B)** distal colon sections from control, sham, KO, DEX, and KO + DEX-treated mice (scale bar = 50 μm, 20× magnification). Muscle layer thickness **(C)**, mucosal width **(D)**, Alcian blue staining density/Area (%) **(E)**, and histological score **(F)** were quantified. Data expressed as mean ± SEM, *p < 0.05, **p < 0.01, ***p < 0.001, ****p < 0.0001 compared with *Win/Win* sham-treated mice; ^##^*p* < 0.01, ^###^*p* < 0.001, ^####^*p* < 0.0001 compared with *Win/Wt* control mice.

H&E and Alcian blue staining were performed on cross-sections of the distal colon from control, sham, KO-, DEX-, and KO + DEX-treated mice to evaluate the efficacy of KO treatment on gross morphological damage and goblet cell density (Figures 2A,B). Thickening of the muscle layer is considered a histological indicator of inflammation. Our results show that the thickness of colonic muscle layer (total thickness of longitudinal and circular muscles) was greater in sham*-*treated mice (123.15 ± 13.72 μm, *n =* 7) compared to control mice (42.14 ± 5.87 μm, *p* < 0.001, *n =* 7, Figure 2C). Muscle thickness was reduced following treatments with KO (74.01 ± 7.45 μm, *p* < 0.05), DEX (66.86 ± 8.33 μm, *p* < 0.05), and KO + DEX (56.28 ± 5.15 μm, *p* < 0.01) in *Win*/*Win* mice (*n =* 8/group). A significant increase in colonic mucosal width was also observed in colon sections from sham-treated mice (420.84 ± 16.23 μm, *n =* 7) compared to control mice (164.79 ± 18.52 μm, *n =* 8, *p* < 0.0001, Figure 2D). Treatments with KO (337.74 ± 27.54 μm, *p* < 0.05), DEX (273.71 ± 21.70 μm, *p* < 0.01), and KO + DEX (218.01 ± 26.40 μm, *p* < 0.0001) decreased the thickness of the colonic mucosal layer (*n =* 7/group) in *Win*/*Win* mice.

Quantification of Alcian blue-positive area revealed a marked reduction in mucin content in sham-treated *Win*/*Win* mice (0.41 ± 0.08%) compared to controls (1.54 ± 0.21%, *p* < 0.0001, *n =* 7/group) (Figures 2E). Treatments with KO or DEX partially restored mucin content, as indicated by increased Alcian blue-positive area (KO: 0.76 ± 0.12%, NS; DEX: 0.62 ± 0.08%, NS, *n =* 6/group), with KO + DEX showing the greatest recovery (1.14 ± 0.19%, *p* < 0.05, *n =* 6) when compared to sham group.

Sham-treated mice exhibited severe mucosal damage characterized by leukocyte infiltration, crypt elongation, abscesses, goblet cell loss, and crypt architectural distortion, resulting in a higher total histological score (9.61 ± 0.75, *n =* 9, *p* < 0.0001) when compared to control mice (1.64 ± 0.22, *n =* 7, Table 2, Figure 2F). Repair of the colonic architecture was observed in colon sections from KO (6.19 ± 0.56, *p* < 0.01), DEX (6.30 ± 0.35, *p* < 0.01), and KO + DEX-treated (5.33 ± 0.77, *p* < 0.001, *n =* 7/group) mice when compared to *Win*/*Win* sham group mice, although the histological scores in KO, DEX, and KO + DEX treatment groups remained higher than in control mice.

**Table 2:** Assessment of parameters for histological scoring.

Parameter	Control (*n* = 9)	Sham (*n* = 7)	KO (*n* = 7)	DEX (*n* = 7)	KO + DEX (*n* = 7)
Aberrant crypt architecture (0–3)	0.63 ± 0.15^***^	1.50 ± 0.14	1.09 ± 0.06	0.96 ± 0.08^*^	0.86 ± 0.09^**^
Increased crypt length (0–3)	0.00 ± 0.00^***^	1.35 ± 0.27	0.45 ± 0.22^*^	0.87 ± 0.25#	0.50 ± 0.25
Goblet cell depletion (0–3)	0.24 ± 0.13^***^	2.57 ± 0.28	2.16 ± 0.30^###^	2.33 ± 0.26^###^	1.70 ± 0.33^###^
General leukocyte infiltration (0–3)	0.58 ± 0.14^***^	2.34 ± 0.14	1.57 ± 0.17^###**^	1.40 ± 0.15^##**^	1.49 ± 0.16^###**^
Crypt abscesses (0–3)	0.00 ± 0.00^*^	0.37 ± 0.17	0.03 ± 0.03^*^	0.00 ± 0.00^*^	0.06 ± 0.06
Epithelial damage and ulceration (0–3)	0.20 ± 0.08^***^	1.45 ± 0.12	0.89 ± 0.16^##*^	0.74 ± 0.13^#**^	0.72 ± 0.16^#**^
Total histological scoring	1.64 ± 0.22^***^	9.61 ± 0.75	6.19 ± 0.56^###**^	6.30 ± 0.35^###**^	5.33 ± 0.77^###***^

Data expressed as mean ± SEM. ^*^p < 0.05, ^**^p < 0.01, ^***^p < 0.001 compared to *Win/Win* sham-treated mice. ^#^*p* < 0.05, ^##^*p* < 0.01, ^###^*p* < 0.001 compared to *Win/Wt* control mice.

### 3.3 Krill oil treatment restored barrier function and suppressed immune cell infiltration in *Winnie* mice

**Figure 3:**
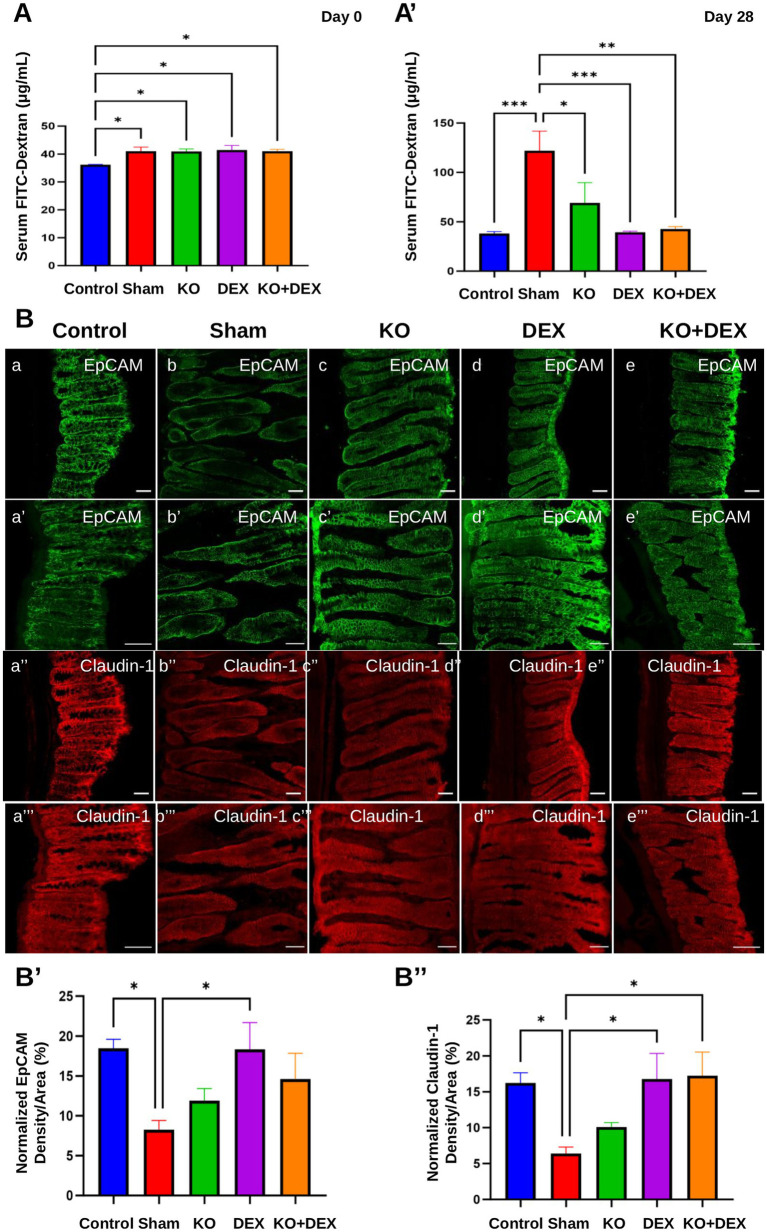
The effects of KO, DEX, and KO + DEX treatments on colonic permeability in *Winnie* mice. Colonic paracellular permeability in *Winnie* mice was assessed using FITC-dextran on day 0 **(A)** and day 28 **(A’)**. **(B)** The effects of KO, DEX, KO + DEX treatment on tight junction protein expression were analyzed in the distal colon of *Winnie* mice. Mucosal epithelial cells were labeled using the anti-EpCAM (green) antibody, anti-Claudin-1 antibody (red), and nuclei marker DAPI (blue) in the colon cross-sections. Representative immunofluorescence images of EpCAM **(a,a’)** and Claudin-1 **(a,”a”’)**. Images were acquired at 20 × (scale bar: 100 μm; **a**, **a”**) and 63 × (scale bar: 30 μm; **a’**,**a”’**) magnification. Quantification of EpCAM **(B’)** and Claudin-1 **(B”)** immunoreactivity normalized to mucosal width of the colon sections from control, sham-, KO-, DEX-, and KO + DEX-treated mice (*n* = 6-7/group). Data expressed as mean ± SEM. *p < 0.05, **p < 0.01, ***p < 0.001 compared with *Win/Win* sham-treated mice.

The effects of KO, DEX, and KO + DEX treatments on colonic paracellular permeability in *Winnie* mice were assessed using FD4 (fluorescein isothiocyanate-dextran, 4 kDa), a fluorescent tracer of paracellular permeability, and IHC analyses of the tight junction protein Claudin-1 and the epithelial adhesion molecule EpCAM (Figure 3). Prior to the treatments (day 0), the serum FD4 levels in all *Win/Win* groups was higher than in *Win/Wt* mice (Figure 3A). The serum FD4 levels across the intestinal mucosa increased further in *Win/Win* sham-treated mice (121.91 ± 19.93 μg/mL, *n =* 7) on day 28 compared to control mice (38.16 ± 1.94 μg/mL, *p <* 0.001, *n =* 8, Figure 3A’). After 28 days of treatments with KO, DEX, and KO + DEX, serum FD4 levels were significantly reduced in *Win/Win* mice (KO: 68.97 ± 20.60 μg/mL, *p <* 0.05; DEX: 39.42 ± 1.05 μg/mL, *p <* 0.001; KO + DEX: 42.69 ± 2.48 μg/mL, *p <* 0.01, *n =* 8/group), close to or comparable to control levels.

To explore the potential mechanisms underlying KO-induced reductions in intestinal permeability, IHC was performed in cross-sections of the distal colon to assess IR of EpCAM and Claudin-1 (Figures 3B–B”). Both EpCAM and Claudin-1-IR were reduced in colon sections from sham-treated (EpCAM: 8.26 ± 1.15%, *n =* 7, Claudin-1: 6.39 ± 0.91%, *n =* 6) when compared to control mice (EpCAM: 18.46 ± 1.13%, *n =* 8; Claudin-1: 16.22 ± 1.42%, *n =* 7, *p <* 0.05 for both). Treatment with DEX elevated EpCAM expression in colon sections from *Win/Win* mice (18.33 ± 3.36%, *p <* 0.05, *n =* 7) to the levels similar to those in control mice, while KO (11.91 ± 1.49%, *n =* 7) and KO + DEX (14.61 ± 3.23%, *n =* 7) treatments tend to enhance the expression of EpCAM in *Win/Win* mice but no statistically significant difference from sham-treated mice was observed. Administration of DEX (16.77 ± 3.55%, *p <* 0.05) and the combination of KO + DEX (17.24 ± 3.29%, *p <* 0.05) increased Claudin-1 expression in *Win/Win* mice to levels comparable to those in *Win/Wt* mice. Although not statistically significant, KO (10.07 ± 0.64%, *n =* 6) tended to elevate Claudin-1 expression compared with sham-treated mice.

**Figure 4:**
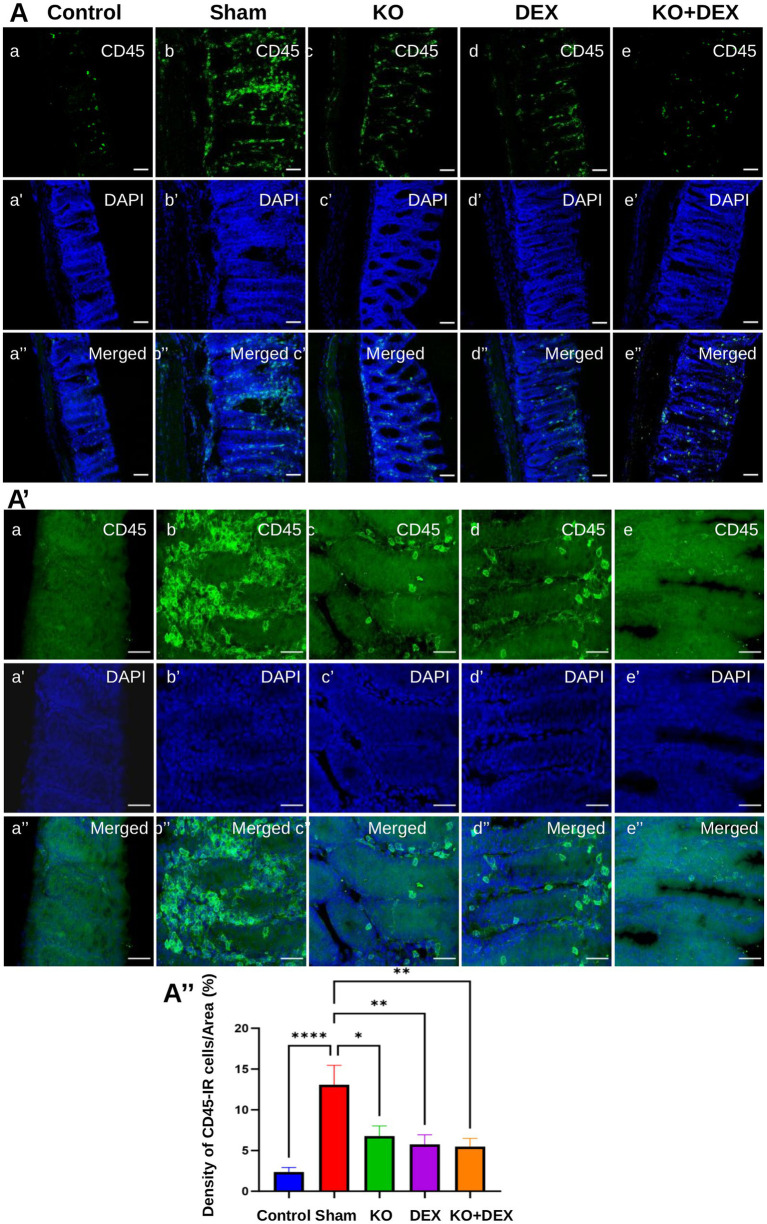
Krill oil attenuates CD45+ immune cell infiltration in the colonic mucosa of *Winnie* mice. **(A,A’)** CD45+ leukocytes were labeled using a leukocyte marker anti-CD45 (green) antibody in the colon cross-sections. Mucosal epithelial cells are labeled with a nuclear marker DAPI (blue). Representative overview images **(A)** were acquired at 20x magnification (scale bar = 100 μm), and high-magnification images **(A’)** were acquired at 63x magnification (scale bar = 30 μm). **(A”)** Quantitative analyses of CD45-IR cells in the mucosa of the colon sections (*n* = 7/group). *p < 0.05, **p < 0.01, ****p < 0.0001 compared with *Win/Win* sham-treated mice.

To evaluate the effects of KO, DEX, and KO + DEX treatments on colonic immune cell infiltration, immunohistochemistry was performed using the pan-leukocyte marker CD45. CD45-immunoreactive (CD45-IR) cells were quantified as the percentage of CD45-IR area relative to the total 2mm^2^ mucosal area (Figures 4A–A”). Sham-treated *Win*/*Win* mice exhibited significantly elevated CD45 + cell density (12.08 ± 2.36%) compared to controls (2.39 ± 0.54%, *p* < 0.0001, *n =* 7/group). Treatment with KO (6.78 ± 1.34%, *p* < 0.05), DEX (5.76 ± 1.18%, *p* < 0.01), and KO + DEX (5.49 ± 1.00%, *p* < 0.01) significantly reduced CD45 + cell infiltration in the colonic mucosa.

### 3.4 Transcriptomic profiling revealed CAMs suppression and barrier restoration

**Figure 5:**
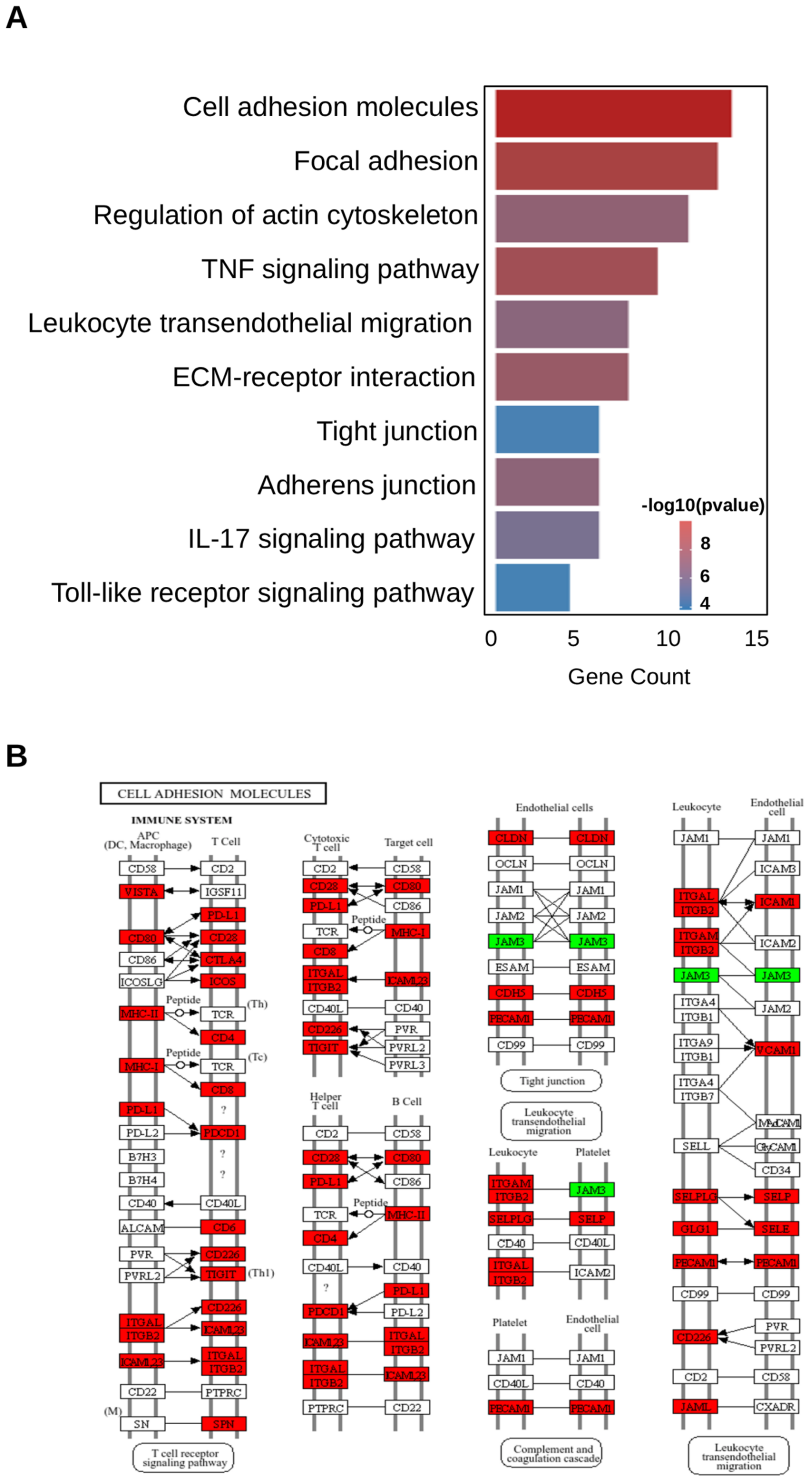
Pathview-based mapping of CAMs pathway reveals treatment-induced transcriptional changes in *Winnie* mice. **(A)** Top 10 enriched KEGG pathways among barrier-related DEGs in *Winnie* mice. Circle size indicates the number of genes involved, and colour intensity reflects adjusted *p* values. **(B)** Differentially expressed genes (DEGs) identified between *Win*/*Win* and *Win*/*Wt* mice were mapped onto the CAMs pathway (mmu04514). Node colours represent log₂*FC*: red indicates upregulation, green indicates downregulation.

To further explore transcriptomic pathways associated with the positive effects of KO, DEX, and KO + DEX treatments on intestinal barrier, we performed transcriptomic profiling of colonic tissues from *Winnie* mice. KEGG pathway enrichment analysis of DEGs overlapping with the barrier-associated gene set revealed significant enrichment in the cell adhesion molecules (CAMs) pathway (mmu04514), with the highest adjusted *p*-value among all tested pathways (Figure 5A). To visualize treatment-induced transcriptional changes within this pathway, we applied the Pathview R package (v1.48.0) to map log₂ fold changes for *Win*/*Win* sham-treated mice relative to the *Win*/*Wt* controls (Figure 5B), KO, DEX, and KO + DEX treated groups relative to the *Win*/*Win* sham-treated group (Supplementary Figure S2).

In *Win*/*Win* sham-treated mice, CAMs pathway analysis revealed transcriptional dysregulation involving genes encoding key immune activation and adhesion molecules. Upregulated genes include *Cd80*, *Cd28*, *Ctla4*, *Icos*, *Pdcd1*, *Cd274* (PD-L1), *H2-K1*, *H2-Ab1*, *Cd4*, *Cd8*, *Itgal*, *Itgb2*, *Icam1*, *Selp*, *Sele*, *Selplg*, *Cd6*, *Cd226*, *Tigit*, *Cldn*, *Cdh5*, *Itga6*, and *Pecam1* compared to *Win*/*Wt* control group. These transcriptional changes suggest enhanced leukocyte adhesion, antigen presentation, and T cell activation. In contrast, downregulation of *Jam3* suggests impaired epithelial integrity (Figure 5B). Following 28 days of KO supplementation, transcriptomic profiling revealed reduced immune activation and modest improvement in barrier-associated gene expression. KO treatment was associated with lower expression of *Icam1*, *Selp*, and *Sele*, suggesting a potential link to reduce leukocyte-endothelial adhesion (Supplementary Figure S2). DEX treatment produced broader immunosuppressive effects, downregulating multiple genes encoding immune activation markers (*H2-K1*, *Cd80*, *Cd226*, *Pdcd1*, *Icam1*, *Itgb2*, *Itgam*, *Selplg*, *Selp*, *Sele*) (Supplementary Figure S2). The KO + DEX combination further reduced expression of immune activation markers (*H2-K1*, *Pdcd1*, *Icos*, *Ptprc*, *Tigit*) and adhesion-related genes (*Icam1*, *Selp*, *Sele*) (Supplementary Figure S2).

### 3.5 Epithelial barrier-associated gene expression supports complementary barrier repair

**Figure 6:**
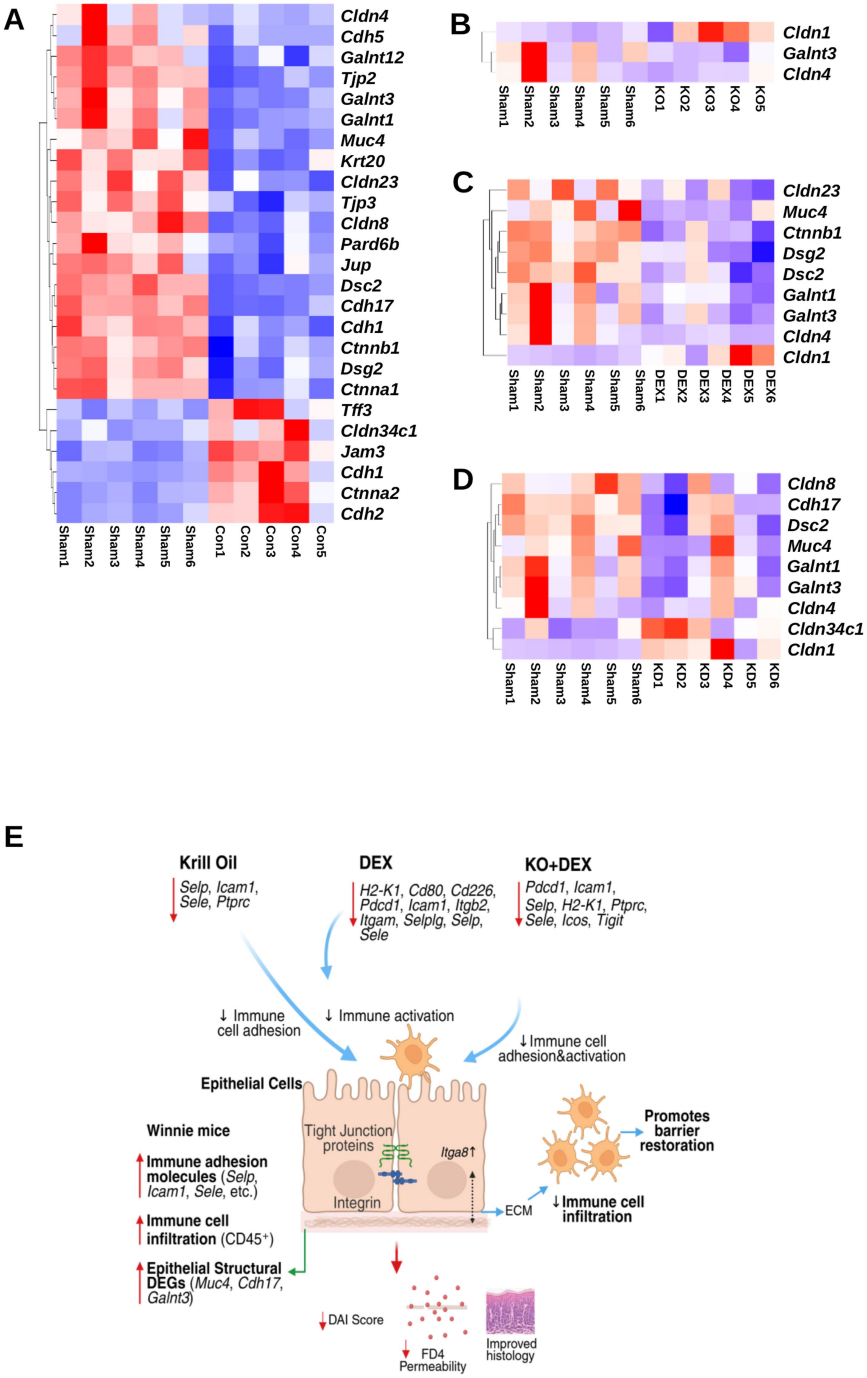
Heatmap of differentially expressed genes (DEGs) associated with barrier-associated structural genes in the colon tissues of *Winnie* mice. **(A)** Comparative heatmap showing expression profiles of barrier-associated structural DEGs in *Win*/*Win* and *Win*/*Wt* mice (*n* = 6/group). **(B–D)** DEGs in colon samples from *Win*/*Win* mice treated with KO, DEX, and KO + DEX compared with sham-treated *Win*/*Win* mice (*n* = 6). Each column represents an individual colon sample, and each row corresponds to a DEG. Gene expression levels are presented as *Z*-score distributions (−2 to +2) based on counts per million (CPM). Upregulated genes are shown in red; downregulated genes, in blue **(E)** Mechanistic overview of CAM and barrier gene modulation by KO, DEX, and KO + DEX in *Winnie* mice.

To complement the CAMs pathway investigation, we further examined DEGs overlapping with a curated set of core epithelial structural and permeability-associated genes. Heatmaps were generated to visualize sample-level expression patterns (*Z*-score range: −2 to +2) across treatment groups (Figures 6A–D), enabling a more granular assessment of epithelial barrier modulation.

Compared to *Win*/*Wt* controls, the *Win*/*Win* sham-treated group exhibited 19 upregulated and 6 downregulated genes within this epithelial structural and permeability-associated genes set, including tight junction components (*Cldn1*, *Cldn4*, *Cldn8*, *Cldn23*, *Cldn34c1*, *Tjp2*, *Tjp3*, *Jam3*), adherens junction molecules (*Cdh1*, *Cdh2*, *Cdh5*, *Cdh17*), desmosomal proteins (*Dsg2*, *Dsc2*, *Jup*), cytoskeletal anchors (*Ctnna1*, *Ctnna2*, *Ctnnb1*, *Krt20*), glycosylation enzymes (*Galnt1*, *Galnt3*, *Galnt12*), and epithelial polarity and barrier regulators (*Pard6b*, *Tff3*, *Muc4*), indicating widespread transcriptional disruption of epithelial integrity (Figure 6A). KO treatment increased the expression of *Cldn1*, and decreased *Galnt3* and *Cldn4*, implicating selective modulation of tight junction components and mucin-type O-glycosylation pathways (Figure 6B). DEX treatment modulated a broader set of 9 DEGs, including *Cldn23*, *Muc4*, *Ctnnb1*, *Dsg2*, *Dsc2*, *Galnt1*, *Galnt3*, *Cldn4*, and *Cldn1*, reflecting its more extensive impact on epithelial architecture and signaling (Figure 6C). The combined KO + DEX intervention further expanded the DEG profile, with 9 genes affected: *Cldn8*, *Cdh17*, *Dsc2*, *Muc4*, *Galnt1*, *Galnt3*, *Cldn4*, *Cldn34c1*, and *Cldn1* (Figure 6D), indicating an additive modulation of epithelial junctional components and mucin-type O-glycosylation pathways.

**Figure 7:**
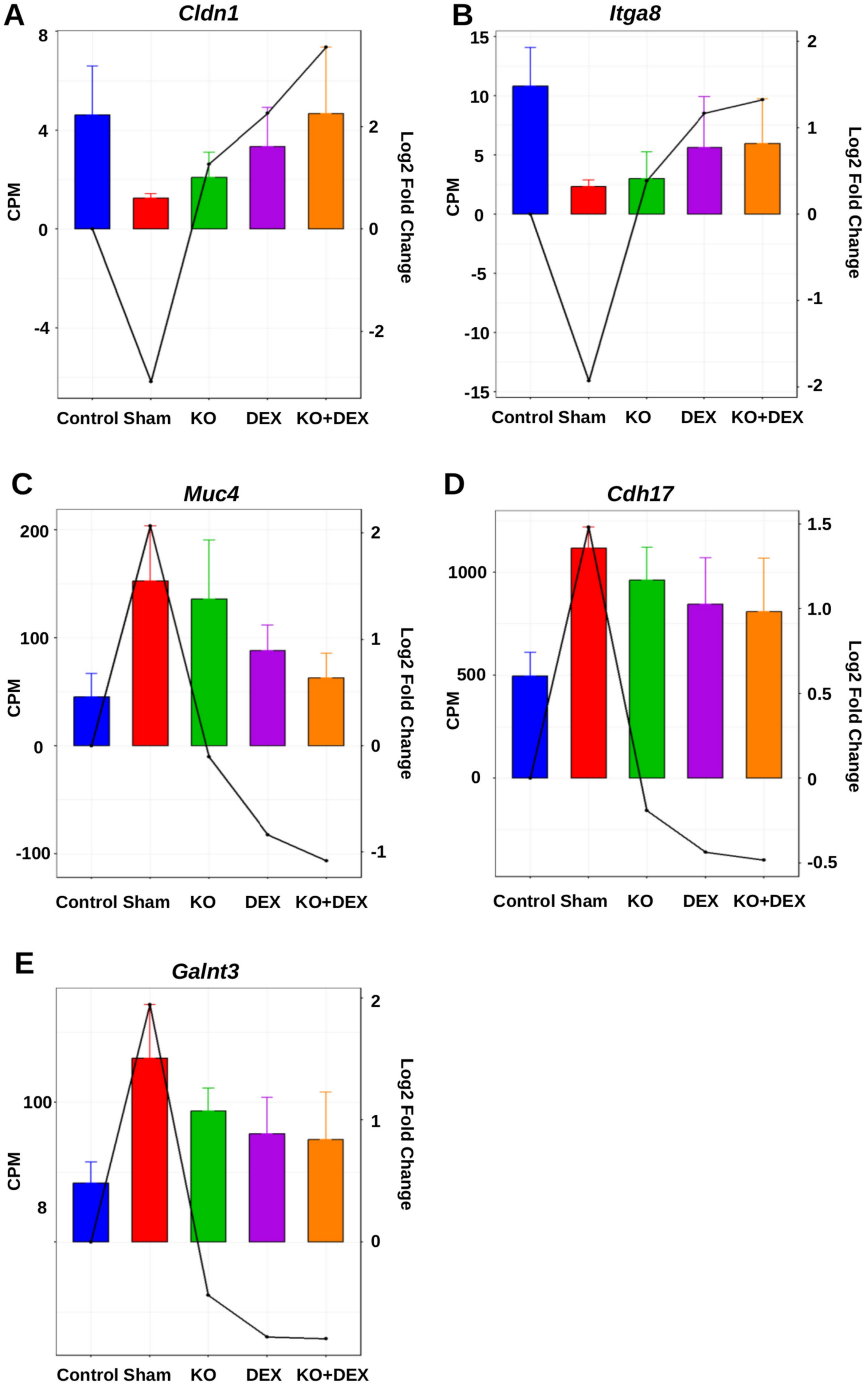
Expression profiles of barrier-associated core structural genes. Transcriptomic analysis was performed on distal colon samples from five experimental groups: *Win*/*Wt* control, *Win*/*Win* sham, KO-treated, DEX-treated, and KO + DEX-treated *Win*/*Win* mice. Genes shown include *Cldn1* **(A)**, *Itga8* **(B)**, *Muc4* **(C)**, *Cdh17* **(D)**, and *Galnt3* **(E)**. Bar graphs represent differential gene expression in counts per million (CPM; left y-axis), while overlaid line plots indicate log₂ fold changes (log₂FC; right y-axis) relative to *Win*/*Win* sham group. Bars represent mean ± SD.

To further refine our understanding of treatment-specific transcriptional effects on epithelial barrier restoration, we examined the expression of five representative genes: *Cldn1*, *Itga8*, *Muc4*, *Cdh17*, and *Galnt3,* which were selected based on their functional relevance and differential regulation across treatment groups (Figures 7A–E). *Cldn1* was downregulated in sham-treated mice but progressively restored by KO, DEX, and KO + DEX treatments, with KO + DEX group achieving near-control levels. *Cdh17, Galnt3,* and *Muc4* were upregulated in sham-treated mice and partially normalized by KO + DEX treatment. Notably, *Itga8* was suppressed in sham-treated mice and selectively upregulated by KO + DEX treatment, reinforcing epithelial-extracellular matrix (ECM) interactions critical for barrier integrity.

## 4 Discussion

This study demonstrates that dietary supplementation with KO effectively ameliorates clinical symptoms and promotes intestinal barrier restoration in a mouse model of spontaneous chronic colitis. The main outcomes of this study include: (1) KO treatment significantly alleviated colitis-associated clinical symptoms, including rectal bleeding, inflammation-induced weight loss, and diarrhea, while improving stool consistency and reducing histopathological severity; (2) KO treatment mitigated enhanced colonic permeability, mildly upregulated Claudin-1 and EpCAM expression, and decreased immune cell infiltration; and (3) Transcriptomic analysis revealed associations between KO treatment and changes in epithelial barrier-related and CAM-related gene expression. These results indicate the promising potential of KO for IBD therapy. In addition, combined treatment with KO and DEX showed increased efficacy in mitigating colitis-associated clinical symptoms and enhanced restoration of epithelial architecture and barrier function in *Winnie* mice compared to DEX monotherapy.

Since sex hormones have been reported to modulate the metabolism of n-3 PUFAs, as well as the anti-inflammatory efficacy of glucocorticoids (26–29), we performed sex-stratified analysis of DAI scores. Our data showed consistent treatment effects across groups, but no significant sex differences were observed within any treatment group. Given that there were only 10 mice in each treatment group and subgroup sample sizes were limited, further sex-stratified examination with larger animal numbers would be useful to validate the current findings. The histomorphological characteristics of IBD are marked by significant morphological damage in the colon, including extensive (sub)mucosal infiltration of inflammatory cells, crypt abscesses, ulcerations, mucin depletion and goblet cell loss, crypt architectural distortion and loss, and thickening of the muscularis mucosae (30, 31). Previous studies have demonstrated that *Winnie* mice exhibit several signs of colonic mucosal damage, including thickening of the muscle and mucosal layers, depletion of goblet cells, heightened intestinal permeability, and increased immune infiltrate through the colonic wall due to a point mutation in the *Muc2* mucin gene (15, 32). Consequently, *Winnie* mice show clinical symptoms similar to those of human IBD, including chronic diarrhea, ulcerations, and rectal bleeding, establishing this model to be highly representative of IBD. In this study, clinical symptoms and inflammation-induced gross morphological damage in the distal colon, including crypt architectural distortion, crypt abscesses, goblet cell loss, and thickening of the muscularis and mucosa, were evident in *Win/Win* sham-treated mice, consistent with previous studies (16, 20). KO, DEX, and KO + DEX treatments attenuated clinical symptoms and changes to the colonic architecture. KO supplementation showed comparable efficacy to DEX in alleviating clinical signs, such as rectal bleeding, diarrhea, and body weight loss in *Win/Win* mice. The combination of KO and DEX treatments resulted in a slightly enhanced effect on DAI compared to KO and DEX treatments alone. Similarly, KO combined with DEX showed a greater effect in attenuating aberrant crypt architecture and goblet cell depletion compared to KO or DEX alone. This implicates the potential role of KO as an adjunctive treatment agent for IBD. Although colonic architecture was restored following KO, DEX, and KO + DEX treatments compared to sham-treated *Win/Win* mice, histological scores remained higher than control *Win/Wt* mice. These findings suggest that extending the treatment duration beyond 28 days may further enhance mucosal recovery.

It is well established that intestinal epithelial barrier dysfunction contributes to IBD pathogenesis, with tight junction protein dysregulation implicated in increased permeability (33). In this study, we report increased colonic permeability and reduced expression of Claudin-1 and EpCAM, in distal colon sections from *Win/Win* sham-treated mice. Transcriptomic analysis revealed dysregulation of Claudin family genes in *Winnie* mice, with *Cldn1* and *Cldn1*5 downregulated consistent with inflammation-induced barrier disruption and *Cldn4* upregulated, reflecting a compensatory repair response (34). Previous studies in *Muc2*^−^/^−^ mice similarly reported downregulation of *Cldn1* (35). Given that EpCAM plays a crucial role in forming a functional barrier in the intestinal epithelium by recruiting Claudins to tight junctions (36), reduced expression of EpCAM in *Win/Win* mice colons correlates to the decreased Claudin-1-IR demonstrated in this study. The loss of tight junction proteins and depletion of goblet cells contributed to subsequent increase of intestinal permeability in *Win/Win* mice. This study revealed that KO, DEX, and KO + DEX treatments effectively reduced intestinal permeability, as evidenced by decreased serum FD4 levels. In our study, KO treatment showed a modest, non-significant increase in Claudin-1 and EpCAM expression, whereas DEX significantly upregulated both proteins to near-control levels. These results suggest that KO and DEX may exert their protective effects on the intestinal barrier through distinct regulatory mechanisms or signaling pathways.

To investigate the molecular mechanisms underlying KO- and DEX-mediated barrier restoration, we performed transcriptomic profiling of colonic tissues, focusing on genes associated with epithelial integrity. KEGG pathway enrichment revealed significant modulation of the cell adhesion molecules (CAMs) pathway (mmu04514), which plays a central role in leukocyte trafficking, epithelial junctional stability, and neuroimmune signaling (37). Several key adhesion molecules, such as *Icam1*, *Selp*, *Sele*, and *Ptprc*, play pivotal roles in the pathogenesis of colitis by mediating leukocyte recruitment, endothelial activation, and mucosal immune infiltration (38). In our study, KO treatment selectively suppressed the expression of genes encoding these adhesion molecules (*Icam1*, *Selp*, *Sele*, *Ptprc*), suggesting a potential role in the attenuation of immune cell recruitment and restoration of epithelial-immune homeostasis. CD45 immunostaining also confirmed high level of immune cell density in the colonic mucosa of *Win/Win* sham-treated mice, which was decreased following treatment. This targeted modulation contrasts with DEX’s broader immunosuppressive profile, which downregulated a wider array of immune markers (e.g., *H2-K1*, *Cd80*, *Cd226*, *Pdcd1*, *Itgb*, *Selplg*), consistent with glucocorticoid-mediated transcriptional repression via NF-κB and AP-1 inhibition (39). However, DEX’s systemic immunosuppression is associated with adverse effects such as bone loss, mood disturbances, and increased infection risk (39). Notably, KO + DEX did not simply amplify DEX-like immunosuppression. Instead, it generated a complementary and more balanced transcriptional response, with selective suppression of adhesion-related genes (*Icam1*, *Selp*, *Sele*) and only a limited subset of immune activation markers (*Pdcd1*, *Icos*, *Tigit*). This pattern reflects targeted modulation rather than global immune repression, aligning with the enhanced barrier restoration observed in this group.

KO treatment was associated with altered expression patterns of structural and permeability-related genes, including *Cldn1*, *Cldn4*, *Galnt3*, and *Muc4*, compared with sham-treated mice. Claudins regulate paracellular permeability and maintain mucosal homeostasis (40). *Galnt3* encodes GalNAc-T3, a glycosyltransferase that initiates mucin-type O-glycosylationis essential for mucus layer biosynthesis. Impaired *Galnt3* expression disrupts mucin glycosylation, compromises mucus barrier function, and increases susceptibility to colitis (41, 42). These findings suggest that KO may selectively enhance junctional architecture and mucin-type glycosylation, two critical determinants of epithelial barrier function and mucus layer integrity.

Furthermore, activation of β-catenin (*Ctnnb1*) signaling enhances WNT ligand secretion by macrophages, promoting intestinal stem cell regeneration and alleviating inflammatory injury (43). Desmoglein-2 (*Dsg2*), a cadherin-type desmosomal adhesion molecule, is essential for maintaining intestinal barrier properties. Reduced *Dsg2* protein levels and altered desmosome ultrastructure have been implicated in IBD pathogenesis (43, 44). DEX treatment modulated a broader set of junctional and cytoskeletal genes, including *Ctnnb1*, *Dsg2*, and *Muc4*, reflecting its capacity to induce epithelial remodelling. The KO + DEX group exhibited the most extensive transcriptional normalization, with restoration of *Cldn8*, *Cdh17*, *Galnt3*, and *Itga8*, indicating that combined treatment more effectively reinforced epithelial polarity, glycosylation, and ECM anchoring. Collectively, these findings suggest that KO enhances DEX’s barrier-stabilizing effects through complementary modulation of structural and immune pathways (Figure 6E). Future investigations using qPCR, Western blotting, or ELISA to validate the transcriptomic data generated from this study will be helpful to better understand the molecular mechanisms underlying the positive effects of KO. This dual targeting of epithelial integrity and immune regulation positions KO as a promising adjunctive agent that may potentiate therapeutic efficacy of corticosteroid monotherapy. Further studies focusing on additional molecular targets and signaling pathways associated with the beneficial role of KO in chronic colitis, such as cytokine-cytokine receptor interaction, TNF signaling and downstream NF-κB-mediated inflammatory pathways will be useful to understand how KO promotes epithelial repair and restores mucosal homeostasis. In addition, animal studies and clinical trials are required to assess whether KO can reduce corticosteroid dosage, mitigate steroid-associated toxicity, as well as enhance mucosal healing in IBD patients.

## 5 Conclusion

This study demonstrates that dietary KO supplementation exerts significant therapeutic effects in a mouse model of spontaneous chronic colitis, comparable to DEX treatment and enhanced when used in combination. KO treatment alleviated clinical symptoms, restored epithelial architecture, and improved intestinal barrier function. Transcriptomic profiling revealed associations between KO treatment and the upregulation of barrier-associated genes (*Cldn1*, *Cldn4*, *Galnt3*) and downregulation of genes encoding pro-inflammatory adhesion molecules (*Icam1*, *Selp*, *Sele*, *Ptprc*), indicating reinforcement of tight junction integrity and attenuation of immune cell infiltration. Notably, KO + DEX combination therapy further restored epithelial polarity, mucin-type glycosylation, and extracellular matrix anchoring, as well as normalization of *Ctnnb1*, *Dsg2*, *Muc4*, and *Itga8* expression. These findings highlight KO’s efficacy as a useful therapeutic agent or an adjunct to enhance corticosteroid efficacy in a preclinical model. Clinical trials are warranted to validate it’s therapeutic potential in IBD patients.

## Data Availability

The datasets presented in this study can be found in online repositories. The names of the repository/repositories and accession number(s) can be found below: https://www.ncbi.nlm.nih.gov/,—BioProject: PRJNA1444759—BioSample: SAMN56769883—SAMN56769910 (28 samples)—SRA: SRR37941623—SRR37941650 (28 runs).

## Supplementary material

SUPPLEMENTARY FIGURE S1 Study design and treatment regime.

SUPPLEMENTARY FIGURE S2 Pathview visualization of the Cell Adhesion Molecules (CAMs) pathway showing treatment‑induced transcriptional changes in *Winnie* mice. **(A-C)** Differentially expressed genes (DEGs) were mapped onto the CAMs pathway (mmu04514) for each treatment comparison: **(A)** KO vs *Win*/*Win*; **(B)** DEX vs *Win*/*Win*; **(C)** KO+DEX vs *Win*/*Win*. Node colours represent log₂ fold‑change values, with green indicating downregulation relative to the *Win*/*Win* sham group.
